# Beyond the positive glow: how school climate inadvertently challenges mental health by modifying the relationship between BMI and depression in adolescents

**DOI:** 10.3389/fpsyg.2025.1663969

**Published:** 2025-11-05

**Authors:** Ling Jiang, Qianyun He, Chaonan Chen

**Affiliations:** ^1^School of law and public administration, Yibin University, Yibin, China; ^2^School of Government, Beijing Normal University, Beijing, China

**Keywords:** underweight, overweight, depression, body esteem, school climate

## Abstract

**Introduction:**

Adolescence is a critical period for developing body esteem, shaped by physical changes and societal aesthetics, significantly impacting mental health. The relationship between body mass index (BMI) and depression warrants further exploration of mediating and moderating factors.

**Methods:**

A sample of 1,639 rural Chinese adolescents completed questionnaires. Mediation and moderation analyses were performed using SPSS 19.0.

**Results:**

The results showed that body esteem mediated the relationship between underweight status and depression, as well as the relationship between overweight status and depression among boys. Among girls, body esteem only mediated the relationship between underweight status and depression. Moreover, a positive school climate diminished the protective role of body esteem against depression for both boys and girls. It also strengthened the positive association between underweight status and body esteem for boys but weakened the negative association between underweight status and depression for girls.

**Discussion:**

Although a positive school climate correlates with better mental health, it may inadvertently reinforce harmful body esteem standards, highlighting the need for gender-specific approaches in school policies and interventions.

## 1 Introduction

Obesity and depression are two prominent global health issues that contribute significantly to the medical and economic burden worldwide (Tyrrell et al., 2019). Depression affects over 264 million adults globally, with an estimated prevalence of 4.7% and an annual incidence of 3% (Kessler et al., 2015). Similarly alarming statistics apply to obesity, which affects more than 650 million adults worldwide, with at least 2.6 million deaths attributed to obesity each year (Blasco et al., 2020). The risk of both obesity and depression increases significantly during the transition from childhood to adolescence (Fryar et al., 2018; Rao and Chen, 2009). Puberty introduces a series of dramatic physical, social and cognitive changes that not only trigger adjustments in diet, physical activity, and lifestyle habits, thereby increasing the risk of obesity, but also mark a sensitive period for processes associated with the onset, persistence, and recurrence of depressive episodes (Rao and Chen, 2009; Song et al., 2023). Obese adolescents are more likely to experience depression, contributing to poorer academic performance, increased peer victimization, and a higher incidence of reported suicide attempts (Nemiary et al., 2012).

The association between obesity and depression in adolescents has been confirmed at the cross-sectional level (Sutaria et al., 2019). However, unlike the association found for obesity, evidence regarding the relationship between body mass index (BMI) and depression remains inconsistent (He et al., 2022). Currently, three main conclusions have emerged concerning the relationship between BMI and depression. The first suggests a dose–response relationship, where higher BMI levels are associated with increased depression (He et al., 2022; Luppino et al., 2010). The second identifies a U-shaped curve, indicating that individuals with either very high or very low BMI are at an increased risk of depressive symptoms (Badillo et al., 2022). The third finding aligns with the “fat and jolly” hypothesis, which proposes that overweight and obese individuals are less likely to experience depression (Palinkas et al., 1996). This phenomenon may be more common in certain cultures where overweight and obese individuals receive greater social support and acceptance (Dearborn et al., 2018; Revah-Levy et al., 2011). However, it is essential to consider some socio-demographic factors such as cultural environment, age, and gender when interpreting the relationship between BMI and depression.

### 1.1 BMI and depression among rural Chinese adolescents

The prevalence of underweight and overweight/obesity among rural Chinese adolescents has increased in recent years, driven by complex factors (He et al., 2016). The intense examination culture in China reduces opportunities for physical activity and increases sedentary behavior, particularly among students in rural areas who are competing for limited slots in academic high schools (Salmon, 2018). Additionally, the boarding system and socio-economic changes in rural China have exacerbated nutritional imbalances among adolescents (Luo et al., 2009; Zhang et al., 2021). As issues related to abnormal weight become more prevalent, their impact on adolescent depression has been widely confirmed by numerous studies and should not be overlooked (Chen et al., 2024). When gender is added to the consideration, the situation becomes even more complex. Several studies have found that overweight and obesity are associated with increased odds of depression in Chinese adolescents, with this association possibly being stronger in boys (Zhang et al., 2021; Zhao et al., 2019). In contrast, studies conducted in developed countries often conclude that the relationship between overweight/obesity and depression is stronger in girls (Blaine, 2008; Merikangas et al., 2012). The traditional preference for boys in Chinese culture likely contributes to the higher prevalence of depression among overweight/obese boys compared to girls during adolescence (Wang et al., 2014; Zhao et al., 2019). Although the preference for boy has diminished in recent generations, it may still influence attitudes and expectations toward rural boys (Zhao et al., 2019).

However, the psychological effects of being underweight have received relatively little attention, and the findings of the few available Chinese studies have been mixed. A large-scale national survey demonstrated a link between perceived weight and depressive symptoms among Chinese middle school students. The results indicated that perceived underweight was associated with increased depressive symptoms in boys, but not in girls (Zhang et al., 2021). Another study reported that being underweight increased the risk of emotional, behavioral, and overall difficulties in boys, but decreased the risk of these problems in girls (Ren et al., 2018). These mixed findings suggest that the relationship between BMI and adolescent depression is likely influenced not only by the weight itself but also by additional factors. Internal perceptions and external environmental influences are key variables that may help explain these inconsistencies. Drawing on Ecological Systems Theory (Bronfenbrenner, 1979), adolescents’ mental health can be understood as the result of continuous interactions between individual characteristics and multiple layers of the surrounding environment. Specifically, adolescents’ internal perceptions of their body image, such as how they view their weight relative to others (Jim E Nez-Morcillo et al., 2024), reflect individual-level processes, whereas external environmental factors, including cultural norms, social expectations, and school climate (Choukas-Bradley et al., 2022), represent broader contextual influences. Together, these factors shape how adolescents perceive themselves and, in turn, affect their mental health. Therefore, future research should adopt an ecological perspective to examine how internal and external factors interact across different environmental levels to influence the relationship between BMI and depression in adolescents.

### 1.2 The mediating role of body esteem

The relationship between BMI and adolescent depression is complex and can be understood within an ecological framework. At the individual level, one potential mediator in this relationship is body esteem, which is defined as an individual’s attitudes, evaluations, and feelings about their body (Williams et al., 2013). It is a component of self-concept that includes positive or negative perceptions of one’s appearance, shape, size, and overall body esteem (Williams et al., 2013). At the macro system level, broader sociocultural values and media representations of ideal body types shape shared expectations about appearance. Within this ecological context, the social culture provides a framework for understanding how the internalization of social ideals and the resulting body esteem mediate the relationship between BMI and depression (Lowy et al., 2021). The Western beauty ideal for girls emphasizes a tall, thin, and curvy physique, while boys are expected to be muscular but slim (Kable, 2023). When an individual’s BMI does not match this internalized ideal, it can lead to body dissatisfaction and low body esteem (Tsiantas and King, 2001). Similarly, evidence from China indicates that higher BMI among girls and lower BMI among boys are associated with poorer body image and lower self-evaluations, reflecting internalized gendered body ideals (Liu et al., 2023). Longitudinal findings indicate that these effects are reciprocal but predominantly unidirectional, showing that BMI predicts subsequent declines in body satisfaction rather than the reverse (Blundell et al., 2024). At the individual level, this negative body esteem represents an internal vulnerability factor that heightens the risk of depressive symptoms. Adolescents who internalize an observer’s view of the physical self can result in body shame, appearance anxiety, and a reduced awareness of the body’s internal state (Fredrickson and Roberts, 1997). These consequences of self-objectification have been linked to mental health risks, including depression (Fredrickson and Roberts, 1997). Evidence from Malaysia demonstrated a significant negative correlation between body esteem and depression, as well as between body esteem and eating disorders (Manaf et al., 2016). Furthermore, girls were found to be more vulnerable to the negative effects of body esteem than boys (Manaf et al., 2016). Longitudinal studies have also shown the significant predictive power of body esteem on changes in depressed mood in both boys and girls (Holsen et al., 2001). In conclusion, body esteem issues may be both psychological and societal, and they represent a potential factor in explaining the relationship between BMI and depression (Kable, 2023).

### 1.3 The moderating role of school climate

From an ecological systems perspective, school environment may be a critical window when assessing the relationship between BMI, body esteem, and depression in adolescents. Adolescents spend the majority of their day at school, and the school climate, defined as the quality and character of school life, has a significant impact on their mental health and wellbeing (Cohen et al., 2009). A safe, supportive, and healthy school climate can greatly influence adolescent development, promoting academic achievement, wellbeing, and self-esteem (MacNeil et al., 2009; Way et al., 2007), with positive effects that may last for years (Borgonovi and Pal, 2016). This supportive and inclusive school climate can create a “herd effect,” where students struggling with body esteem and BMI are surrounded by peers with positive and optimistic perspectives on these issues. As a result, these students can develop stronger social and emotional skills and form supportive social relationships (Leurent et al., 2021). Conversely, punitive and stigmatizing environments can exacerbate the negative effects of BMI on mental health (Aldridge and McChesney, 2018).

Ecological theory also emphasizes that the same environment can have differential effects on individuals depending on how they experience and interpret it (Bronfenbrenner, 1979), a principle exemplified by the health context paradox. It points to the phenomenon that in healthy environments with lower rates of bullying, the few children who suffer from bullying will instead experience more adjustment problems, such as heightened levels of depressive symptoms (Li et al., 2024; Xiong et al., 2023). While this paradox has been primarily examined in the context of bullying, it raises the question of whether its underlying principles might apply to other areas as well. Research indicates that an engaging, inclusive, and learner-centered school climate can foster motivation and engagement by stimulating curiosity among some students (Bosacki et al., 2023; Marchante et al., 2022). However, the same school climate, shaped by both students and teachers, may also induce anxiety and worry, as it emphasizes learner control and intellectual freedom in the classroom (Marchante et al., 2022). Furthermore, adolescents who perceived a high level of informality and familiarity in their school relationships have been found to exhibit higher levels of anxiety and depression (Kasen et al., 2004). This may be due to the fact that adolescents’ perceptions of positive social relationships and support can result in varying self-evaluations (Ioannou et al., 2019). At times, this positive atmosphere and relationship may activate positive schematics, while at other times, it may convey messages that threaten self-esteem, triggering conflict and comparisons with others (Choenarom et al., 2005). These findings illustrate the interactive and dynamic nature of ecological systems, where the quality of the environment interacts with individual differences to shape adolescents’ mental health trajectories.

Given the transition during adolescence from the family to other significant figures as models of behavioral norms, school experiences and climates, particularly those involving relationships with peers and teachers, may increasingly impact the relationship between adolescents’ BMI, body esteem and depression (Kasen et al., 2009). A study involving 4,086 students in grades seven and eight found that loneliness increased in grade eight for both girls and boys with higher weight in schools where weight monitoring was more prevalent in grade seven (Juvonen et al., 2019). Similar effects were observed for low self-esteem among girls (Juvonen et al., 2019). This evidence supports two reasonable hypotheses: first, that a positive school climate moderates the relationship between adolescent BMI and depression, and second, that it also moderates the relationship between BMI and body esteem. In addition, a nurturing school environment plays a vital role in promoting acceptance and support among peers. When students feel valued and appreciated, they are less likely to succumb to negative self-comparisons and the associated risk of depression (de Arellano et al., 2023). Nevertheless, a paradox may arise in closely knit school communities where peer visibility intensifies social comparison (Watt, 2003). Adolescents may experience pressure to fit in with their peers, which can amplify feelings of inadequacy in their body esteem (Leurent et al., 2021). Thus, this study hypothesizes that school climate plays a moderating role between body esteem and depression.

### 1.4 Gender difference

Adolescence is widely recognized as a critical period for the emergence of gender differences in depression prevalence (Salk et al., 2017). During this stage, girls are approximately twice as likely as boys to experience depression (Salk et al., 2017). This disparity is believed to result from a complex interplay of biological, social, and cultural factors (Albert, 2015). Although BMI and body esteem concerns are common across genders, the ideal body esteem differs between boys and girls. In China, two traditional cultural preferences for body shape coexist. One preference idealizes a slim female physique, historically deemed attractive (Leung et al., 2001). Specifically, features such as a slender waist, a slim figure, and a fragile-looking body have been particularly prized, with practices like foot binding and girdling once being prevalent (Leung et al., 2001). This preference does not extend to males, for whom strong muscles and a large body size are advantageous, a standard reinforced by mass media. For instance, the term “gao fu shuai” refers to an ideal male partner who is tall, wealthy, and handsome, rather than slim (Sun, 2017). Additionally, adolescent boys and girls exhibit differing patterns of development and socialization, which may influence their perceptions of the school climate (Wang and Dishion, 2012). Boys, for example, may have a less favorable view of the school climate than girls, possibly due to differing expectations related to relationships and gender norms (Wang, 2009). When examining the impact of school climate on problematic behavior, the influence of school climate appears to be more significant for boys (Kuperminc et al., 1997). These inconsistent findings, coupled with gender differences in how BMI and body esteem relate to depression, suggest the need for further research to investigate gender differences in the moderating role of school climate in the relationship between these variables.

### 1.5 The present study

Adolescent development is shaped by the continuous interaction between individual characteristics and multiple environmental systems. The onset of puberty triggers a rapid series of changes in weight, height, body shape, body composition, and secondary sexual characteristics. These physical transformations, coupled with increased exposure to cultural ideals of beauty, often lead to heightened body esteem concerns. During adolescence, individuals are particularly prone to engaging in self-comparison and internalizing societal beauty standards, which can negatively impact their body esteem. With the acceleration of globalization, the influence of Western cultural ideals of thinness as an ideal body standard has become increasingly pronounced among Chinese adolescents, affecting their BMI, body esteem, and mental health.

In rural areas of China, these developmental processes unfold within unique sociocultural contexts characterized by close-knit communities, strong social cohesion, and high levels of social control. Rural adolescents are embedded in environments that emphasize collectivism, conformity, and respect for authority, which can both foster belonging and intensify social comparison and peer evaluation. Moreover, traditional gender norms remain influential: boys are often expected to be strong, capable, and responsible, while girls are encouraged to be modest, self-disciplined, and compliant. These expectations shape how adolescents interpret body ideals and social feedback, influencing both their self-perception and emotional wellbeing. School climate, as a crucial environmental factor within the ecosystem framework, may serve as a moderating variable in the relationships between BMI, body esteem, and depression. The school climate is perceived differently by boys and girls with different BMIs and levels of body esteem, reflecting distinct social norms and expectations that warrant further investigation in a sample of rural Chinese adolescents. By addressing these research gaps and exploring the complex relationships among weight perception, body esteem, and mental health in this population, we can better understand their needs and develop more effective strategies to promote their mental health.

**Figure 1:**
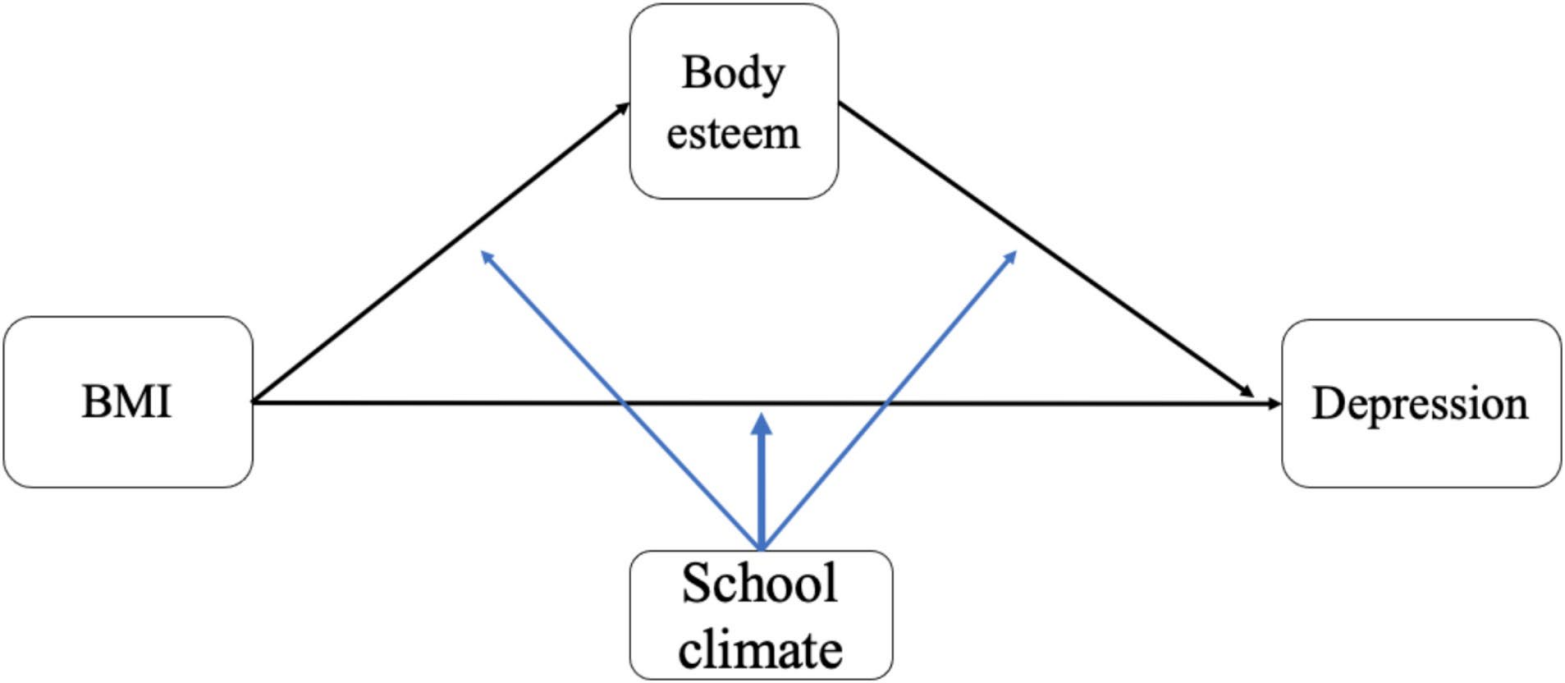
The hypothesized moderated mediation model.

The aims of this study were to (1) examine depression scores and gender differences among adolescents with different BMIs; (2) explore the mediating role of body esteem in the relationship between BMI and depression, as well as gender differences; and (3) investigate the moderating role of school climate in both the direct effect of BMI on depression and the indirect effect of BMI through body esteem among boys and girls. The proposed path model is shown in Figure 1.

## 2 Methods

### 2.1 Participants and procedure

This study was a cross-sectional study conducted in December 2023. Taking into account geographic distribution, time and financial constraints, and sample size considerations, a convenience sampling strategy was employed. Two schools from Beijing and Shandong Province in eastern China, one school from Henan Province in central China, and four schools from Sichuan Province in western China were invited to participate. All of these schools were located in rural areas. The economic development of rural areas in these provinces/municipality is approximately representative of the average level of rural areas across the eastern, central and western regions of China, respectively. In the participating schools, two or three classes were randomly selected from each grade (grades 5–8), and all students in the selected classes were invited to complete a questionnaire regarding their learning, life, and mental health.

Approval for this study was obtained from the University Ethics Committee and the headmasters of all participating schools. Informed consent forms were provided to both students and their parents (or caregivers) to ensure they fully understood the study’s purpose. Our research team was admitted to enter the classroom during school hours to address any questions from the participants. The questionnaires were designed to be completed in approximately 20 min, after which a small gift was distributed to each participant as a token of appreciation. The initial sample consisted of 1,805 rural adolescents, but 1,639 samples were ultimately retained after excluding questionnaires that were carelessly completed or had key items missing.

### 2.2 Measures

#### 2.2.1 BMI

In compliance with Chinese government regulations, all students are required to participate in a physical fitness assessment each academic year. The results of these assessments, including height and weight, are made available to the students. Therefore, we collected self-reported height and weight data from the students and calculated their BMI by dividing their weight by the square of their height (kg/m^2^) in accordance with the National Standard for Student Physical Fitness and Health, which varies by age and gender (Chinese Ministry of Education, 2022). From an ecological–sociocultural perspective, BMI is not merely a biological indicator but a socially constructed attribute that interacts with cultural norms of gender, appearance, and self-worth. Distinguishing between BMI categories allows for a more accurate understanding of how societal expectations and internalized body ideals shape adolescents’ emotional wellbeing. Therefore, BMI was categorized during data analysis in this study. Due to a small number of obese students, BMI was categorized into underweight, normal weight and overweight (including obese).

#### 2.2.2 Depression

Depression was measured using the Patient Health Questionnaire (PHQ). The PHQ is a 9-item instrument based on the DSM-IV diagnostic criteria (American Psychiatric Association, 2022). Each item is scored on a scale from 0 (not at all) to 3 (almost every day). The total score ranges from 0 to 27, with higher scores indicating more depressive symptoms. The validity and reliability of this screening instrument have been well established, and it is widely used for identifying and assessing depressive symptoms, rather than for making clinical diagnoses. The Cronbach’s alpha for the PHQ-9 was 0.869 in this study.

#### 2.2.3 Body esteem

Body esteem was measured using the General Appearance sub-scale (NPSS-G) of the Negative Physical Self Scale (NPSS) (Chen et al., 2006). The NPSS-G consists of five items related to physical appearance, such as “I’m proud of my body,” “I like my body very much,” and “I do not think there is anything about my body that needs to change.” Each item is rated on a scale from 1 (never) to 5 (always). The total score ranges from 5 to 25, with higher scores indicating greater body esteem. The Cronbach’s alpha for the NPSS-G was 0.837 in this study.

#### 2.2.4 School climate

School climate was measured using five items extracted from the China Education Panel Survey (CEPS) and the annual Beijing Haidian District Primary and Secondary School Students’ Mental Health Assessment Programme. These items include “I make friends easily at school,” “I feel a sense of belonging at school,” “other students like me,” “I feel like an outsider at school,” and “I feel lonely at school.” Each item is rated on a scale from 1 (strongly disagree) to 4 (strongly agree). The items “I feel lonely at school” and “I feel like an outsider at school” are reverse-scored. In the calculation of the total school climate score, the values for these two items were reversed. The total score ranges from 5 to 20, with higher scores indicating a more positively perceived school climate. The Cronbach’s alpha for these items was 0.717 in this study.

#### 2.2.5 Confounding variables

This study took the following demographic and socioeconomic characteristics into consideration: Grade level (grade 5/grade 6/grade 7/grade 8), self-rated health (very good/better/average or worse), only child (yes/no), father’s education attainment (junior high school or less/high school/university college and above), mother’s education attainment (junior high school or less/high school/university college and above), family financial situation (better than others/equal to others/worse than others) and self-rated pubertal developmental timing (earlier than others/equal to others/later to others).^1^ While these variables are categorized into broad groups, such classifications are commonly used in adolescent research to simplify data collection and still capture meaningful trends (Izdebski et al., 2025; Anaya, 2024; Wang et al., 2022).

### 2.3 Statistical analyses

Descriptive and correlation analyses were performed using SPSS 19.0. The frequency distributions of the boys’ sample, the girls’ sample and the overall sample across various socio-demographic variables were calculated separately. Means, standard deviations and correlations for body esteem, depression, and school climate were also presented separately for the sub-gender samples. PROCESS 3.5, a freely available SPSS tool developed by Hayes for estimating regression models with mediation and/or moderation effects, was then used to test the mediating role of body esteem in the relationship between BMI (reference group: normal weight) and depression, as well as to examine gender differences in this mediation. Interaction terms (underweight * school climate, overweight * school climate, body esteem * school climate) were created to explore the moderating role of school climate and its gender differences.

## 3 Results

### 3.1 Descriptive statistics and correlation analyses

Data from 1,639 students in grades 5–8 were included in the final analyses, with 868 girls and 771 boys. Table 1 presents the basic characteristics of the sample. The majority of students rated their health as good (68.5%), reported average or good family finances (82.3%), and identified as non-only children (71.3%). Approximately half of the students have parents with an educational level of junior high school or less. A total of 20.1% and 22.9% of students indicated that their physical development was significantly earlier or later than that of their peers, respectively. Additionally, 8.9%, 75.7% and 15.4% of students were classified as underweight, normal weight and overweight, respectively.

**Table 1:** Descriptive analysis of sample characteristics.

Variables	Boy		Girl		Total	
	*N*	%	*N*	%	*N*	%
Self-rated health						
Very good	301	39	340	39.2	641	39.1
Better	237	30.7	244	28.1	481	29.4
Average or worse	233	30.2	283	32.6	516	31.5
Only child						
No	533	69.1	636	73.3	1,169	71.3
Yes	238	30.9	232	26.7	470	28.7
Father’s education attainment						
Junior high school or less	424	55	484	55.8	908	55.4
High school	198	25.7	223	25.7	421	25.7
University college and above	149	19.3	161	18.5	310	18.9
Mother’s education attainment						
Junior high school or less	429	55.6	461	53.1	890	54.3
High school	185	24	217	25	402	24.5
University college and above	157	20.4	190	21.9	347	21.2
Family financial situation						
Better than others	149	19.3	165	19.1	314	19.2
Equal to others	435	56.4	598	69.1	1,033	63.1
Worse than others	187	24.3	103	11.9	290	17.7
Grade						
5th	115	14.9	107	12.3	222	13.5
6th	61	7.9	100	11.5	161	9.8
7th	275	35.7	345	39.7	620	37.8
8th	320	41.5	316	36.4	636	38.8
Self-rated pubertal developmental time						
Earlier than others	149	19.9	169	20.2	318	20.1
Equal to others	428	57.2	476	56.9	904	57.0
later to others	171	22.9	192	22.9	363	22.9
BMI						
Underweight	53	7.3	84	10.3	137	8.9
Normal weight	547	75.6	620	75.9	1,167	75.7
Overweight	124	17.1	113	13.8	237	15.4

The results of the descriptive statistics and correlation analyses for the continuous research variables in this study are presented in Table 2. Pearson correlation analyses showed that body esteem was significantly negatively correlated with both depression and school climate. Additionally, depression was significantly positively correlated with school climate in both samples.

**Table 2:** Descriptive and correlation analyses of continuous variables.

Variables	Boy				Girl					
	*M*	SD	1	2	3	*M*	SD	1	2	3
1 Body esteem	15.33	4.98	1			15.31	4.85	1		
2 Depression	6.01	5.37	−0.371^***^	1		5.88	5.08	−0.364^***^	1	
3 School climate	14.68	3.12	−0.379^***^	0.458^***^	1	14.55	3.16	−0.436^***^	0.500^***^	1

*p < 0.05, **p < 0.01, ***p < 0.001.

### 3.2 The mediation effect of body esteem

The Hayes’s PROCESS (Model 4) was used to examine the mediating role of body esteem in the relationship between BMI and depression. As shown in Table 3, both underweight (*β* = 0.33, SE = 0.74, *p* < 0.05) and overweight boys (*β* = 0.22, SE = 0.51, *p* < 0.05) exhibited significantly higher depression scores compared to their normal weight counterparts. Conversely, underweight girls (*β* = −0.34, SE = 0.60, *p* < 0.01) showed significantly lower depression scores than normal weight girls. After adding body esteem as a mediating variable, the positive correlation between underweight or overweight status and depression was no longer statistically significant among boys. However, the inverse relationship between underweight status and depression remained significant among girls (*β* = −0.27, SE = 0.58, *p* < 0.05). Moreover, body esteem was significantly lower in underweight (*β* = −0.32, SE = 0.68, *p* < 0.05) and overweight boys (*β* = −0.48, SE = 0.47, *p* < 0.001) than in normal weight boys, and significantly higher in underweight girls (*β* = 0.26, SE = 0.54, *p* < 0.05) than in overweight and normal weight girls. Notably, body esteem was significantly negatively related to depression in both boys (*β* = −0.27, SE = 0.04, *p* < 0.001) and girls (*β* = −0.27, SE = 0.04, *p* < 0.001).

**Table 3:** The mediation effect of body esteem on the relationship between BMI and depression.

Gender	Outcomes	Predictors	*R*	*R^2^*	*F*	*β*	SE	*t*	*p*
Boy	Depression		0.41	0.17	11.02^***^				
		BMI (ref: normal weight)							
		Underweight				0.33	0.74	2.43	<0.05
		Overweight				0.22	0.51	2.37	<0.05
	Body esteem		0.44	0.2	13.39^***^				
		BMI (ref: normal weight)							
		Underweight				−0.32	0.68	−2.37	<0.05
		Overweight				−0.48	0.47	−5.14	<0.001
	Depression		0.48	0.23	14.67^***^				
		BMI (ref: normal weight)							
		Underweight				0.25	0.72	1.86	>0.05
		Overweight				0.10	0.50	1.03	>0.05
		Body esteem				−0.27	0.04	−7.19	<0.001
Girl	Depression		0.42	0.17	12.54^***^				
		BMI (ref: normal weight)							
		Underweight				−0.34	0.60	−2.95	<0.01
		Overweight				0.14	0.50	1.44	>0.05
	Body esteem		0.47	0.22	16.44^***^				
		BMI (ref: normal weight)							
		Underweight				0.26	0.54	2.32	<0.05
		Overweight				−0.10	0.46	−1.06	>0.05
	Depression		0.48	0.23	16.65^***^				
		BMI (ref: normal weight)							
		Underweight				−0.27	0.58	−2.41	<0.05
		Overweight				0.11	0.49	1.20	>0.05
		Body esteem				−0.27	0.04	−7.62	<0.001

*p < 0.05, **p < 0.01, ***p < 0.001; the analysis controlled for covariates.

As shown in Table 4, the bootstrap 95% confidence interval (CI) confirmed the significant indirect role of body esteem in the relationship between underweight status and depression (*β* = 0.46, SE = 0.17, 95% CI: 0.15, 0.82), as well as in the relationship between overweight status and depression among boys (*β* = 0.69, SE = 0.18, 95% CI: 0.38, 1.08). Among girls, body esteem showed a significant indirect role only in the relationship between underweight status and depression (*β* = −0.36, SE = 0.17, 95% CI: −0.70, −0.04). Therefore, among boys, body esteem mediated the effects of both underweight and overweight status on depression. Similarly, this mediating role was observed in the relationship between underweight status and depression among girls.

**Table 4:** Simple and conditional median effects of BMI on depression through body esteem at different school climate levels for boys and girls.

Gender	Path	Effect	SE	95% CI	
				LLCI	ULCI
Boy	Total effect: underweight → depression	1.79	0.74	0.35	3.24
	Direct effect: underweight → depression	1.33	0.72	−0.07	2.74
	Indirect effect: underweight → body esteem → depression	0.46	0.17	0.15	0.82
	Conditional indirect effect (low school climate)	0.05	0.27	−0.48	0.62
	Conditional indirect effect (mean school climate)	0.30	0.12	0.10	0.56
	Conditional indirect effect (high school climate)	0.19	0.19	−0.14	0.61
	Total effect: overweight → depression	1.21	0.51	0.20	2.21
	Direct effect: overweight → depression	0.52	0.50	−0.47	1.5
	Indirect effect: overweight → body esteem → depression	0.69	0.18	0.38	1.08
	Conditional indirect effect (low school climate)	0.65	0.22	0.26	1.13
	Conditional indirect effect (mean school climate)	0.40	0.13	0.17	0.68
	Conditional indirect effect (high school climate)	0.14	0.15	−0.11	0.48
Girl	Total effect: underweight → depression	−1.75	0.6	−2.93	−0.59
	Direct effect: underweight → depression	−1.39	0.58	−2.52	−0.26
	Indirect effect: underweight → body esteem → depression	−0.36	0.17	−0.70	−0.04
	Conditional indirect effect (low school climate)	−0.54	0.28	−1.16	−0.05
	Conditional indirect effect (mean school climate)	−0.18	0.10	−0.40	−0.01
	Conditional indirect effect (high school climate)	−0.01	0.05	−0.12	0.08
	Total effect: overweight → depression	0.72	0.50	−0.27	1.71
	Direct effect: overweight → depression	0.58	0.49	−0.37	1.54
	Indirect effect: overweight → body esteem → depression	0.14	0.14	−0.12	0.42

The analysis controlled for covariates.

### 3.3 The moderating effect of school climate

The Hayes’s PROCESS (Model 59) was used to examine the moderating role of school climate. The results of moderated mediation effects are shown in Table 5. Among boys, the interaction between body esteem and school climate had a significant effect on depression (*β* = 0.12, SE = 0.01, *p* < 0.001), as did the interaction between underweight status and school climate on body esteem (*β* = −0.07, SE = 0.22, *p* < 0.001). Among girls, the direct effect of underweight status on depression was significantly moderated by school climate (*β* = 0.07, SE = 0.19, *p* < 0.05). The interaction between body esteem and school climate on depression was also significant (*β* = 0.13, SE = 0.01, *p* < 0.001).

**Table 5:** The moderating effect of school climate on depression.

Gender	Outcomes	Predictors	*R*	*R^2^*	*F*	*β*	SE	*t*	*p*
Boy	Body esteem		0.56	0.31	19.61^***^				
		BMI (ref: normal weight)							
		Underweight				−0.08	0.66	−2.20	<0.05
		Overweight				−0.15	0.44	−4.46	<0.001
		School climate				0.38	0.07	9.55	<0.001
		Underweight * school climate				−0.07	0.22	−2.00	<0.05
		Overweight * school climate				−0.01	0.13	−0.16	>0.05
	Depression		0.53	0.28	14.62^***^				
		BMI (ref: normal weight)							
		Underweight				0.09	0.73	2.45	<0.05
		Overweight				0.03	0.49	0.88	>0.05
		School climate				−0.21	0.08	−4.87	<0.001
		Underweight * school climate				0.07	0.25	1.92	>0.05
		Overweight * school climate				−0.02	0.15	−0.64	>0.05
		Body esteem				−0.19	0.04	−4.86	<0.001
		Body esteem * school climate				0.12	0.01	3.55	<0.001
Girl	Body esteem		0.58	0.33	24.11^***^				
		BMI (ref: normal weight)							
		Underweight				0.06	0.51	2.03	<0.05
		Overweight				−0.03	0.43	−0.87	>0.05
		School climate				0.42	0.06	11.0	<0.001
		Underweight * school climate				−0.04	0.18	−1.31	>0.05
		Overweight * school climate				−0.05	0.13	−1.37	>0.05
	Depression		0.57	0.33	20.86^***^				
		BMI (ref: normal weight)							
		Underweight				−0.07	0.55	−2.37	<0.05
		Overweight				0.03	0.46	1.08	>0.05
		School climate				−0.35	0.07	−8.47	<0.001
		Underweight * school climate				0.07	0.19	2.31	<0.05
		Overweight * school climate				0.02	0.14	0.61	>0.05
		Body esteem				−0.16	0.04	−4.40	<0.001
		Body esteem * school climate				0.13	0.01	4.15	<0.001

*p < 0.05, **p < 0.01, ***p < 0.001; the analysis controlled for covariates.

**Figure 2:**
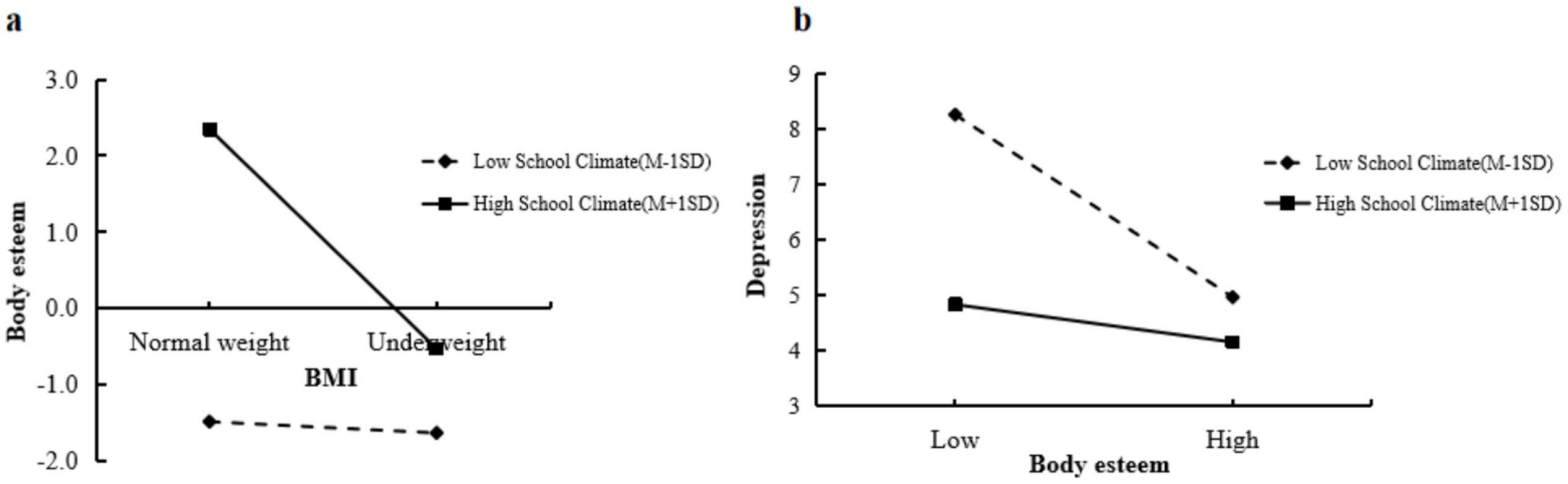
**(a)** Perceived school climate moderates the effect between underweight and body esteem in boys. **(b)** Perceived school climate moderates the effect between body esteem and depression in boys.

**Figure 3:**
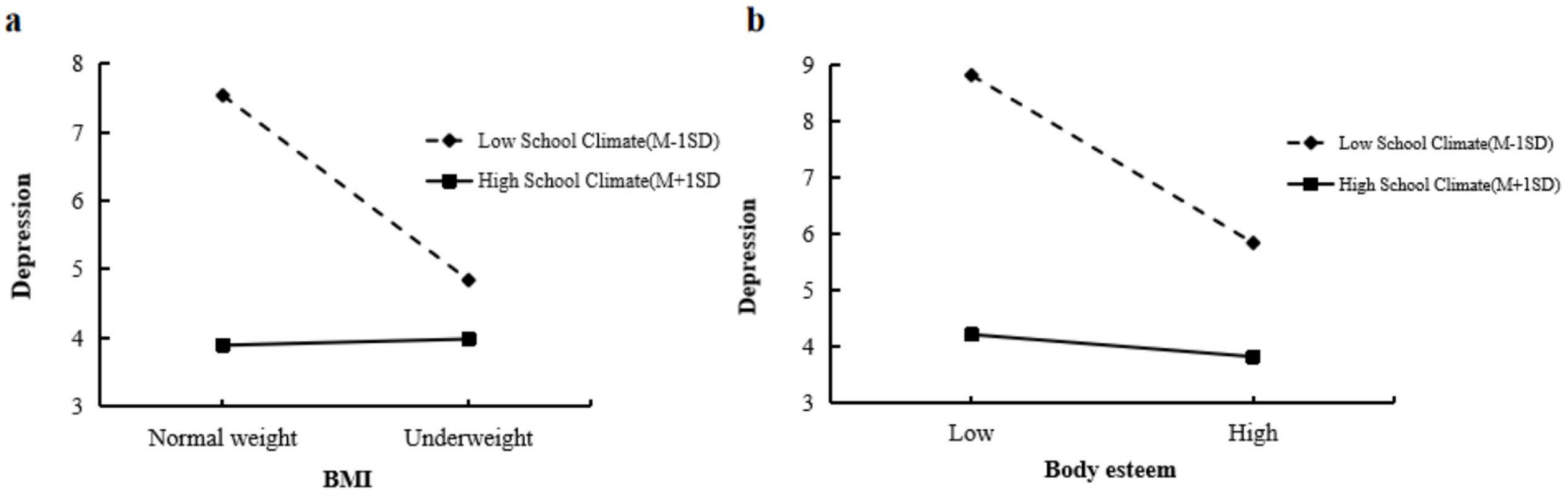
**(a)** Perceived school climate moderates the effect between underweight and depression in girls. **(b)** Perceived school climate moderates the effect between body esteem and depression in girls.

Furthermore, a simple slope analysis was conducted to examine the interaction at low (mean − 1 SD) and high (mean + 1 SD) levels of perceived school climate (see Figures 2, 3). Among boys, the slope of underweight on body esteem was larger at high levels of perceived school climate compared to low levels (see Figures 2a, 3a) indicated that the direct effect of underweight on depression was stronger among girls with low perceived school climate. Both boys and girls with low perceived school climate showed a stronger effect of body esteem on depression compared to those with high perceived school climate (see Figures 2b, 3b). These findings suggest that a positive school climate amplified the negative effect of underweight on body esteem in boys, but weakened the negative effect of underweight on depression in girls. For both boys and girls, a positive school climate weakened the negative relationship between body esteem and depression.

## 4 Discussion

This study examined the effect of BMI on depression and related mechanisms, as well as gender differences in this relationship. The results found that body esteem mediated the relationship between BMI and depression for both boys and girls. Additionally, perceived school climate moderated the indirect pathway for boys, while it moderated both the direct and indirect pathways for girls.

The descriptive and correlational analyses also revealed several noteworthy findings. Boys in rural China exhibited a higher prevalence of overweight, whereas girls were more likely to be underweight, reflecting persistent gendered body norms and sociocultural expectations regarding physical appearance and strength (Wang et al., 2024). However, body esteem was significantly negatively correlated with school climate scores. And a positive correlation was observed between depression and school climate scores, which is theoretically unexpected. One possible explanation, consistent with ecological systems theory (Bronfenbrenner, 1979), is that in tightly connected mesosystems such as rural schools, positive school climates characterized by strong social cohesion and shared norms may also intensify social comparison and conformity pressures, particularly when adolescents fail to meet internalized academic or appearance standards. Such environments, while generally supportive, may inadvertently amplify emotional vulnerability among certain students. This finding aligns with the health context paradox (Li et al., 2024), illustrating that even positive ecological contexts can, under certain conditions, increase psychological distress.

### 4.1 The mediating role of body esteem

Compared to normal weight boys, both overweight and underweight boys exhibited higher depression scores, whereas underweight girls demonstrated lower depression scores. This phenomenon can be attributed to body esteem shaped by social expectations. In rural China, traditional values and the necessity for agricultural labor emphasize physical health and strength in boys (Wang et al., 2024). Consequently, overweight or underweight boys are perceived as deviating from the socially acceptable standards of weight and body esteem. Such deviation may result in exposure to weight-related stigma and a perceived loss of masculinity, particularly during adolescence when strength and vigor are highly valued (Liu et al., 2023). From an evolutionary psychology perspective, physical size and muscularity in males are often associated with perceived dominance, resource-holding potential, and social competitiveness (Frederick and Haselton, 2007). During adolescence, when peer hierarchies intensify, boys with lower body mass may experience diminished perceptions of power and attractiveness, which can translate into lower body esteem and heightened vulnerability to depression (Neves et al., 2017). For underweight girls, the situation differs somewhat. Although underweight girls were less likely to experience depression, approximately 20% of this association was mediated by body esteem. This mediated result confirms the cultural preference for a slim female body, consistent with findings from a study conducted in Hong Kong, China (Lo et al., 2011). Underweight girls may perceive themselves positively, notably if they align with local aesthetic standards that favor slimness. Moderate thinness may also be associated with perceived youthfulness, social desirability and reproductive fitness, traits that are often socially valued (Rodgers et al., 2020). This positive self-perception can reinforce their body esteem, which is especially significant during adolescence, a time when self-image plays a critical role (Dixit et al., 2011). Adolescents frequently utilize maladaptive coping strategies to address distorted body esteem perceptions (Oellingrath et al., 2016). These differences reflect the influence of macro system-level cultural norms interacting with individual perceptions of body image, illustrating how societal values and gender expectations shape the relationship between BMI, body esteem, and depression. Understanding the unique pressures boys and girls face regarding body esteem can inform the development of gender-specific programs designed to enhance body esteem and reduce the risk of depression.

### 4.2 The moderating role of school climate

The present study also identified gender-based difference in the moderating effects of school climate on the relationship between BMI, body esteem, and depression. The tripartite influence model, which posits that school climate is a critical factor in explaining body esteem dissatisfaction, suggests that social influences, including peer, familial, and media pressures, compel individuals to conform to culturally defined aesthetic standards (Stojcic et al., 2020). Typically, a positive school climate is expected to have a buffering effect, reducing the risk of depression by fostering a supportive environment and enhancing overall wellbeing (Bosacki et al., 2023). However, the present study found that for both boys and girls, a positive school climate diminished the protective role of body esteem in preventing depression. Since body esteem is related to how adolescents perceive their worth, in low school climate environments, a lack of support and feelings of disconnection from others may amplify the negative emotional consequences of low body esteem, thereby increasing vulnerability to depression (Stea et al., 2024). In contrast, the social pressures and emotional demands inherent in supportive school environments may shift the psychological mechanisms, making body esteem less of a direct predictor of depression. In this context, social comparison and the need for belongingness may become more prominent, potentially leading to increased feelings of disappointment and frustration (Hansen et al., 2021).

Secondly, among boys, a positive school climate served to reinforce the positive correlation between underweight status and depression. This finding reaffirms the reverse buffering effect of a positive school climate. In educational settings that prioritize a positive school climate, young boys with low body mass may feel increasing pressure to maintain a robust and masculine self-image, especially when interacting with peers whose weight matches societal expectations of confidence and security (Cohen et al., 2009). As a result, they may experience heightened feelings of inadequacy or depression. Furthermore, all participants in this study were from rural Chinese schools, which are typically small enough to provide a high level of social support and social control, fostering a close-knit community with shared values (homogeneity) (Watt, 2003). While such social control has the potential to mitigate deviant behavior among adolescents in the community, it must be approached with caution, as excessive control may be counterproductive, potentially increasing the risk of depression and suicide among boys compared to girls in these schools (Watt, 2003). According to self-determination theory, excessive control undermines adolescents’ basic psychological needs for autonomy and competence, which in turn increases their vulnerability to depression (Ryan et al., 2022). Within ecological systems framework, this can be interpreted as a meso system-level effect, where the tight interconnections among family, school, and community create strong normative pressures that may restrict individual variability, particularly in contexts that emphasize conformity and gendered expectations (Bronfenbrenner, 1979). During puberty, messages surrounding the muscle ideal disseminated rapidly among peers in highly homogeneous environments, and then underweight boys begin to internalize the appearance ideal, exposing them to a higher risk of body esteem dissatisfaction.

Thirdly, among girls, a positive school climate attenuated the negative correlation between underweight status and depression, potentially protecting against distorted perceptions of weight. In recent years, greater gender empowerment and the growing public awareness in China of the physical and psychological risks associated with extreme dieting and excessive thinness have gradually challenged the traditional thin ideal among women (Hanson et al., 2024). Consequently, it is plausible that positive school environments foster tolerance and respect for the diversity of girls’ body sizes, helping underweight girls feel safe and valued. These findings highlight the interactive nature of ecological systems, demonstrating how the interplay between individual factors and contextual influences shapes adolescents’ emotional wellbeing. Understanding these cross-system dynamics provides a more comprehensive ecological explanation of gender differences in the relationship between BMI, body esteem, and depression.

### 4.3 Limitations and implications

The present study had several limitations. First, while the investigation provides valuable insights into the relationships between BMI, body esteem, depression, and school climate, its cross-sectional design limits the ability to draw definitive causal conclusions. Further exploration through longitudinal research is crucial. Second, self-reported BMI in adolescents poses significant challenges due to the influence of body esteem ideals, possibly leading to overestimation of height and underestimation of weight. Although the height and weight data used in this study were collected during mandatory annual physical fitness assessments in schools, where students typically remember their most recent measurements clearly, the self-reported nature of BMI may still be affected by these biases. Third, the schools were selected through convenience sampling, which may limit the representativeness and external validity of the findings. Future studies should adopt more systematic or stratified sampling methods to enhance generalizability. Furthermore, measuring school climate sub-dimensions is essential for understanding the complex relationships between school climate and student outcomes. Finally, while the school climate measure has been validated in several large-scale surveys in China, the Cronbach’s alpha of 0.717 in this study may reflect a limitation of the sample size. Future studies with larger sample sizes could improve reliability, and further validation could enhance the tool’s cross-cultural applicability.

Despite the above limitations, the study has important implications. This study sheds light on the mediating role of body esteem in the relationship between BMI and depression, encouraging educators to work to create positive educational experiences that help adolescents develop positive body esteem. A further key finding is that a positive school climate does not necessarily amplify the protective effects of body esteem. Indeed, in some cases, it may even undermine its role in preventing depression. This suggests the need for a more nuanced understanding of how school climate interacts with adolescents’ individual characteristics as well as for the exploration of ways to optimize school environments. For example, peer-led body esteem programs can empower students to challenge appearance-related stereotypes and support one another in building healthy body perceptions. Teacher training on weight-related stigma and inclusive classroom practices help reduce subtle biases and social comparison in the classroom. In addition, social–emotional learning activities that emphasize self-acceptance and coping with social comparison can further enhance resilience. Given the observed gender differences, gender-sensitive interventions should address the distinct pressures on boys and girls, such as promoting healthy muscularity ideals among boys and challenging the thin ideal among girls. By embedding these strategies within an overall supportive and inclusive school climate, educators can mitigate the paradoxical effects of positive climates and foster environments that genuinely promote mental health and body esteem.

## 5 Conclusion

The study examined the underlying mechanism between BMI and depression in adolescents. The findings indicated that body esteem mediated the relationship between BMI (underweight and overweight) and depression in boys, as well as between underweight status and depression in girls. Additionally, the moderating effects of school climate on the direct and indirect pathways differed by gender. Specifically, a positive school climate was found to reduce the protective role of body esteem in preventing depression for both boys and girls. Similarly, a positive school climate was observed to strengthen the positive association between underweight status and body esteem for boys, while weakening the negative association between underweight status and depression for girls. Although a positive school climate is generally associated with better mental health outcomes, it does not uniformly enhance protective factors like body esteem. In some cases, it may inadvertently contribute to mental health challenges by reinforcing harmful body esteem standards or social comparisons among students.

## Data Availability

The raw data supporting the conclusions of this article will be made available by the authors, without undue reservation.

## Footnotes

^1^Father’s education attainment, mother’s education attainment, family financial situation and self-rated pubertal developmental timing were classified on a 5-point scale. This approach of grouping participants into three categories helps ensure a more balanced and interpretable dataset.
